# Translational Insights into Exercise-Induced Protective Adaptations in 5XFAD Mice and Middle-Aged Amateur Sportsmen

**DOI:** 10.3390/antiox15060698

**Published:** 2026-05-31

**Authors:** Pau Garcia-Baucells, Clara Bartra, Sara Sarroca, Filipa Reinoite, Júlia Senserrich, Rubén Corpas, Christian Griñán-Ferré, Coral Sanfeliu

**Affiliations:** 1 Departament de Neurociències i Terapèutica Experimental, Institut d’Investigacions Biomèdiques de Barcelona (IIBB), CSIC, 08036 Barcelona, Spain; pgarcia4@researchmar.net (P.G.-B.); clara.bartra@iibb.csic.es (C.B.); sarasarroca@gmail.com (S.S.); filipa.reinoite@itqb.unl.pt (F.R.); julia.senserrich@unican.es (J.S.); rubencorpas@gmail.com (R.C.); 2 Institut Hospital del Mar d’Investigacions Mèdiques (IMIM), 08003 Barcelona, Spain; 3 Independent Researcher, 08015 Barcelona, Spain; 4 ITQB NOVA, NOVA University of Lisbon, 2780-157 Lisbon, Portugal; 5 Instituto de Biomedicina y Biotecnología de Cantabria (IBBTEC, UC-CSIC), Centro de Investigación Biomédica en Red de Salud Mental (CIBERSAM), 39011 Santander, Spain; 6 Department of Pharmacology, Toxicology and Therapeutic Chemistry, University of Barcelona, Av. Joan XXIII, 27-31, 08028 Barcelona, Spain; christian.grinan@ub.edu; 7 Institute of Neurosciences of the University of Barcelona, 08035 Barcelona, Spain; 8 Centro de Investigación en Red, Enfermedades Neurodegenerativas (CIBERNED), Instituto de Salud Carlos III, 28029 Madrid, Spain

**Keywords:** physical exercise, antioxidant genes, mitochondria complexes, proteasome activity, anti-aging and anti-neurodegeneration effects, 5XFAD mice, human peripheral blood, rugby players, Alzheimer’s disease, healthy brain aging

## Abstract

The increase in antioxidant defenses mediated by physical exercise signaling is proposed to be a protective factor against brain aging and neurodegeneration. However, the processes involved, particularly the response of senescence markers and cell fitness status in the context of Alzheimer’s disease (AD) pathology, remain unclear. We analyzed male and female 5XFAD transgenic AD mice subjected to 6 months of voluntary wheel running using molecular and behavioral techniques. Levels of mRNA of selected genes, mitochondrial complex proteins, and proteasomal function, were analyzed in the cerebral cortex or hippocampus. In an exploratory translational approach, proteasomal dynamics- and senescence-related genes were analyzed in peripheral blood from middle-aged amateur sportsmen. Physical exercise increased expression of antioxidant genes and modulated epigenetic genes in 5XFAD male and female mice. An increase in protein levels of hippocampal mitochondrial complexes CIII and CV was also induced in males. Both exercised 5XFAD mice and veteran sportsmen showed improved proteasomal status and decreased expression of senescence genes. Exercised mice showed memory and behavior preservation, as well as increases in brain metabolic fitness and gene modulation. These changes may contribute to the neuroprotective effect of physical exercise.

## 1. Introduction

The contribution of a physically active lifestyle to maintaining an active mind and a physiologically healthy brain are undisputed [1]. Individuals with greater engagement in physical activity showed lower risk of suffering age-related memory loss and brain pathologies such as Alzheimer’s disease (AD) [2,3,4,5]. For instance, in an 8-year prospective study of elderly women, walking approximately 28 km per week was associated with a 7% lower decline in cognitive performance compared with participants who walked only 1 km per week [5]. Furthermore, in a cohort of middle-aged and older adults followed for 6.9 years, the hazard ratio for incidental dementia was reduced to 0.49 with an optimal daily step count just under 10,000 step (≈7 Km) [3]. Physically active AD patients were more protected against clinical progression of AD than sedentary ones, despite similar amyloid burden [6,7]. Furthermore, physical exercise can induce slight cognitive improvement in the initial stages of AD, mainly with regard to the instrumental activities of daily living [8]. Clinical and epidemiological studies are ongoing to discern the most suitable window for and training program against cognitive decline in old age [9,10]. Translational studies are needed to understand the underlying mechanisms favoring the best brain functionality to counteract brain senescence and neurodegeneration. Different AD mouse models have been subjected to various types of physical exercise for either short periods (1 month) or long periods (3 to 9 months). Overall, the outcomes were largely positive, showing decreased behavioral and psychological symptoms of dementia (BPSD)-like behaviors, preserved memory function, and reduced AD-like pathological markers [11,12,13,14]. Recent reports in 5XFAD mice, which develop early AD pathology with high amyloid burden and neuroinflammation, showed that 3 months of wheel running reduced amyloidosis by improving the meningeal lymphatic system [15] and endoplasmic reticulum autophagia [16] and reduced pathological activation of microglia [17] and astrocytes [18]. All these studies reported cognitive improvement in the exercised 5XFAD mice.

Physical exercise induces the secretion of a variety of signaling molecules in the brain and peripheral organs and tissues, including skeletal muscle [19]. Therefore, chronic exercise generates benefits in the cardiovascular system, the nervous system, and the immune system. Among other neurotrophins, brain-derived neurotrophic factor (BDNF) is considered a first-line driver of exercise benefits in cerebral plasticity [20]. Aerobic use of muscle fibers also generates reactive oxygen species that initiate redox signaling cascades. Therefore, another crucial brain mechanism of exercise is the activation of factors that transduce antioxidant and detoxifying responses that will improve cerebral function [21]. An imbalance in redox homeostasis and consequent oxidative damage to DNA and proteins is the basis of some pathological neuronal changes that are considered hallmarks of aging [22], such as loss of proteostasis and mitochondrial dysfunction. These age-related changes increase the risk of AD. Some advances have also been made in understanding cell senescence in the brain, another cellular hallmark of aging that may increase AD risk [23,24]. Furthermore, physical exercise may induce epigenetic changes that may lead to long-term cognitive improvement [25]. However, how the modulation of these aging hallmarks by exercise may decrease the risk of AD and AD progression severity is poorly known.

Therefore, we aimed to analyze potential mechanisms of neuroprotection against aging and AD progression by long-term aerobic physical exercise in an AD mouse model. We hypothesized that redox signaling induces protective adaptations involving oxidative stress, mitochondrial function, epigenetic regulation, proteostasis, and cellular senescence in the brain. These aging-protective changes would improve cerebral fitness and shield against neurodegeneration. We found mostly confirmative results in male and female mice of the 5XFAD strain. We also analyzed senescence and proteasome gene markers in whole-blood samples from middle-aged amateur rugby players to explore the translational relevance of these anti-aging mechanisms observed in the AD mouse model.

## 2. Materials and Methods

### 2.1. Animals and Physical Exercise Treatment

Male and female AD transgenic mice of the 5XFAD strain were bred and maintained in the Animal House of the University of Barcelona (UB) from progenitors bought from Jackson Lab (Bar Harbor, ME, USA; Ref.: mouse line Tg6799, MMRRC stock #34840). Animals were housed in Makrolon cages (Techniplast, Buguggiatte, Italy) with free access to food and water in a temperature-controlled room (22 ± 2 °C) with a 12 h light/12 h dark cycle. Heterozygous transgenic mice and their wild type (WT) siblings were routinely identified by genotyping an ear biopsy. The phenotype of the 5XFAD model shows early amyloid deposition in the cortical and hippocampus areas, driven by expression of the human APP and PS1 genes bearing familial AD-linked mutations, with a total of three pathological mutations in the APP gene and two mutations in the PS1 gene [26].

Two-month-old 5XFAD male and female mice engaged in voluntary aerobic exercise for 6 months, until they were eight months old. Mice were randomly allocated using the Excel RAND function to either an exercised group (5XFAD-EXE) that was housed in cages with free access to a running wheel (Activity Wheel Cage System for Mice; Techniplast, Buguggiate, Italy) or a sedentary group (5XFAD-SED) that was housed in similar cages without wheel. Two or three mice of the same sex were housed per cage to avoid the harmful effects of prolonged isolation stress [27]. A group of WT male and female mice was included in the study as a sedentary control group (WT-SED). Group size was determined using the resource equation method, which yielded a recommended range of four to seven animals per sex and group. To accommodate potential outliers and the need to allocate tissues across several downstream analyses, we selected the upper end of this range. A total of 42 mice (*N* = 7 males and 7 females per group) were used. At termination, brain tissue from all mice was collected and preserved for molecular analysis. The experiment was conducted in two subsequent single-sex batches. All tissue samples were analyzed simultaneously to avoid batch-related variability. In addition, two weeks before termination, male mice underwent a battery of behavioral tests to confirm the expected positive effects of exercise treatment on memory, and BPSD-like behaviors such as exploratory activity and anxiety behaviors. We also performed a preliminary time-course study using two independent age groups of WT (*N* = 6 per age group, total *N* = 12) and 5XFAD male mice (*N* = 7 per age group, total *N* = 14) to assess basal cognitive and emotional status at 2 and 4 months of age, in comparison with the results from the non-exercised 8-month-old WT and 5XFAD mice included in the main study. These time points correspond to the beginning, intermediate, and termination stages of the study. The mice at termination were at an advanced age corresponding approximately to middle-to-later life in humans [28]. The experimental design and the animal protocols were approved by the UB Ethics Committee of Animal Experimentation (CEEA-UB; protocol code 3933, date of approval 4 February 2018). All procedures were carried out in accordance with Directive 214/97 of the Generalitat de Catalunya and European Union Directive 2010/63/EU for animal experiments. All efforts were made to reduce the number of animals and refine the procedures to minimize suffering. Animals were visually monitored daily in their cages for pain, distress, or endpoints requiring intervention.

### 2.2. Mice Brain Samples

Male and female mice that were part of the main study on brain biomarkers of physical exercise were euthanized at 8 months of age by cervical dislocation after brief anesthesia with isoflurane. The brain was immediately dissected on a cold plate to obtain cerebral cortex and hippocampus samples. Tissue samples were processed immediately or snap-frozen in liquid nitrogen and kept at −70 °C until analysis. The samples were coded, and the molecular analyses described below were performed by a blinded experimenter, with the exception of western blotting. Two- and four-month-old male mice used in the preliminary behavioral study were euthanized using the same procedure, and their brains were dissected and preserved for future studies.

### 2.3. Human Blood Samples

Human peripheral whole-blood samples were obtained from the antecubital vein following overnight fasting. Blood was directly collected in Tempus Blood RNA tubes (Life Technologies, Thermo Fisher Scientific, Waltham, MA, USA), shaken vigorously for 10 s and frozen at −70 °C until RNA extraction. Samples were coded for subsequent blinded analysis.

Human donors were from a male middle-aged physical exercise cohort of amateur rugby players participating in weekly training and veteran competitions (*N* = 24, age: 54.3 ± 6.6 y) and age-matched controls with less than 150 min of weekly leisure-time physical activity (*N* = 25, age: 56.0 ± 5.9 y). The rugby players were long-term practitioners of the sport for an average period of 35 years (range: 7–59 years). All participants had been previously assessed for memory effects, blood redox biomarkers, and gene expression changes induced by long-term leisure physical exercise [29,30]. The study was approved by the Ethics Committee of the Hospital Clínic de Barcelona, Spain (protocol code HCB/2014/0516, date of approval 4 July 2014). Written consent was obtained from all participants. All procedures were conducted in accordance with the 1964 Declaration of Helsinki and its later amendments. Complete demographic data and physical activity rates of human donors are shown elsewhere [29,30].

### 2.4. Real-Time Quantitative Polymerase Chain Reaction

The expression of genes involved in the antioxidant response, epigenetics, and senescence was measured in the cerebral cortex or hippocampus of mice by real-time quantitative polymerase chain reaction (qPCR). The expression of selected senescence- and proteasome-associated genes was determined in human blood samples.

Extraction of RNA from the mouse brain samples was carried out using a mirVana total RNA Isolation Kit (Applied Biosystems, Thermo Fisher Scientific, Waltham, MA, USA) following the manufacturer’s protocol. Human RNA stabilized in Tempus Blood RNA tubes was extracted using a Tempus Spin RNA Isolation Kit (Invitrogen, Thermo Fisher Scientific). Extracted RNA samples were maintained below −70 °C until use. Then, RNA concentration and quality were assessed using a Nanodrop ND-1000 spectrophotometer (Thermo Fisher Scientific). Reverse transcription from RNA to first-strand complementary DNA (cDNA) was performed using a High-Capacity cDNA Reverse Transcription Kit (Applied Biosystems, Thermo Fischer Scientific) and a thermal cycler (FlexCycler, Analytikjena, Jena, Germany). cDNA samples were stored below −20 °C until use. Gene expression was measured using specific commercial TaqMan FAM-labeled probes (Thermo Fisher Scientific) and a CFX96 Real-Time qPCR Detection System (Bio-Rad, Hercules, CA, USA). The list of genes and the corresponding TaqMan Assay ID probes utilized are shown in Supplementary Table S1. Mouse data were normalized to *Actb* and *Tbp*, and human data to *B2M* and *PGK1*. Results were calculated using the comparative cycle threshold (ΔΔCt) method and expressed relative to the mean of male values of the respective control group.

### 2.5. Western Blotting

Relative protein levels of mitochondrial complexes were analyzed in the mouse hippocampus by western blotting. Protein extracts were obtained and electrophoresed, and the protein bands transferred to PVDF membranes, as previously described [31]. The membranes were incubated overnight at 4 °C with Total OXPHOS Rodent WB Antibody Cocktail (1:1000; #ab110413, Abcam, Cambridge, UK). This cocktail contained five monoclonal antibodies that reacted against subunits of the mitochondrial complexes (CI to CV) as follows: CI—NADH:ubiquinone oxidoreductase subunit B8 (NDUFB8), CII—succinate dehydrogenase complex iron sulfur subunit B (SDHB), CIII—cytochrome b-c1 complex subunit 2 (UQCRC2), CIV—cytochrome c oxidase subunit 1 (MTCO1), and CV—ATP synthase subunit 5 (ATP5A). The secondary antibody used was HRP-conjugated sheep anti-mouse (1:12,000; #NA931, Amersham, General Electric, Boston, MA, USA). Protein blots were visualized by enhanced chemiluminescence detection in a Chemidoc™ Imaging System (Bio-Rad, Hercules, CA, USA), and densitometric analysis was performed using Image Lab software (v3.0.1; Bio-Rad). The results were presented as the ratios of CI, CIII, CIV, and CV to CII. This latter complex has been previously shown to be stable and optimal for normalizing mitochondrial protein content in mouse models of AD [32,33].

### 2.6. Proteasome Enzymatic Activity

Proteasomal enzymatic activity was determined in the cerebral cortices of mice. Fresh tissue lysates were obtained and further processed, as previously described [11]. Proteasomal activity was determined using the Proteasome-Glo™ Assay Systems (Promega, Madison, WI, USA). Specific substrates were Suc-LLVY-aminoluciferin for chymotrypsin-like, Z-LRR-aminoluciferin for trypsin-like, and Z-nLPnLD-aminoluciferin for caspase-like activity. Luminescence was measured using an Orion II Microplate Luminometer (Titertek-Berthold, Pforzheim, Germany).

### 2.7. Behavioral and Cognitive Tests

General behavior and cognition were analyzed in the 8-month-old male mice from the main study to confirm the expected positive effects of voluntary wheel-running treatment on BPSD-like behaviors and recognition memory. This testing was also performed in the 2-month-old and 4-month-old male mice included in the time-course study, in a coordinated manner that allowed comparison with sedentary male mice in the main study, to construct a preliminary behavioral phenotype timeline. Therefore, female mouse testing was not performed.

BPSD-like behaviors were evaluated with the open field test, the light–dark box test, and the Boissier’s four-hole-board test. Memory loss or preservation was evaluated with the novel object recognition test (NORT). Behavioral testing protocols and the apparatuses used were described elsewhere [11,34]. The open field test was used to evaluate locomotor activity in a white arena surrounded by walls. This test measures several parameters, including ambulatory movement as an indicator of horizontal exploratory activity and vertical rearing behavior, which reflects the animal drive for exploration and general well-being. The light–dark test was used to assess anxiety-like behavior by quantifying exploration of a brightly illuminated compartment connected to a dark compartment. Although mice are naturally curious and tend to explore the lit area, the aversive illumination induces anxiety-related avoidance. Latency to enter, number of entries, and time spent in the lit compartment are indicators of anxiety-like responses. The four-hole-board test was used to evaluate exploratory drive through head dipping behavior into the holes of the board. Because mice can freely explore the holes without an anxiogenic component, parameters such as the number of head dips, latency to the first hole exploration, and latency to explore all four holes provide insight into exploratory motivation and may reveal reduced exploratory engagement or apathy-like behavior. Together, these three tests provide complementary insight into locomotor exploration, anxiety-like responses, and apathy-related traits, which parallel aspects of BPSDs observed in patients with AD. These BPSD-oriented tests lasted 5 min each and were performed sequentially on different days. Finally, NORT assessed memory by evaluating the mice’s spontaneous tendency to spend more time exploring a novel object when faced with a pair of objects: one previously explored (familiar object) and one new one (novel object). The procedure include 3 consecutive days of habituation to the black, empty arena, the acquisition trial (two identical objects, A + A), a 2 h short-term retention trial (objects A + B), and a 24 h long-term retention trial (B + C). All exploration times lasted 10 min. A discrimination index was calculated. The open field test and NORT performances were video-recorded in a coded file, and the subsequent analysis was performed blinded to the experimental group. However, animals in the light–dark box test and the four-hole-board test were analyzed during the tests, although camera recordings were made for verification if needed. In the main study, one mouse was initially excluded from each group due to poor performance, specifically failing to reach a minimal exploration time in the NORT baseline (<10 s) and other tests. Therefore, *N* = 6 animals were tested for all experimental groups.

### 2.8. Statistics

The results are shown as mean ± SEM. Data distribution was checked with the Shapiro–Wilk normality test. Experiments including males and females were analyzed by two-way ANOVA, with the main factors being sex (male and female) and group (WT-SED, 5XFAD-SED, and 5XFAD-EXE). In the absence of sex effect, the data for males and females were pooled for each group and analyzed by one-way ANOVA. The behavioral results including only sedentary male mice were analyzed by two-way ANOVA with the main factors being age (2, 4, and 8 months) and strain (WT and 5XFAD). Mice and all tissue samples were coded and analyzed in a blinded manner, whenever possible, as described, and were decoded for statistical analysis. Results were considered significant when *p* < 0.05. Post hoc Fisher’s LSD test was performed to compare the means between groups. Human results with two groups were analyzed with Student’s *t*-test. Statistical outliers were identified with Grubbs’ test (α = 0.05) and excluded from the analysis, as indicated in the corresponding figure legends. Due to the eventual reduced sample availability for the diverse molecular analyses or the presence of statistical outliers, we used a final *N* = 4–7 independent units per mouse dataset and N = 24–25 per human dataset; specific numbers are indicated in the figure legends. Data were entered into Microsoft Excel v16.77.1 (Microsoft Corporation, Redmond, WA, USA) for storage and management. Statistical analysis was performed using GraphPad Prism v11.0.0 (GraphPad Software, Boston, MA, USA).

### 2.9. Network and Pathway Module Analysis

Mouse data on mRNA and protein levels were integrated into a hierarchical network analysis to identify core regulatory modules underlying the exercise-induced molecular changes. To increase the power of the analysis and the reliability of the results, data from 5XFAD-EXE males and females were pooled, irrespective of previously reported sex effects, and the changes induced by physical exercise were analyzed relative to WT-SED mice. Therefore, mean values for the WT-SED and 5XFAD-EXE experimental groups were calculated for each molecular target, and fold-change (FC) values were computed as the ratio of group means (5XFAD-EXE/WT-SED). Molecular targets were grouped into four biologically defined pathway modules based on functional annotations and the literature cited in this study: (i) antioxidant response (*Nfe2l2*, *Gpx1*, *Cat*, *Aldh2*, *Sod2*; cerebral cortex); (ii) mitochondrial function (NDUFB8/CI, UQCRC2/CIII, MTCO1/CIV, ATP5A/CV; hippocampus); (iii) epigenetic regulation (*Hdac1*, *Hdac2*, *Hdac5*, *Dnmt1*, *Dnmt3a*, *Dnmt3b*; hippocampus); and (iv) cellular senescence (*Cdkn1a*, *Cdkn2a*, *Trp53*; cerebral cortex). A three-layer directed network was constructed with physical exercise (5XFAD-EXE condition) as the apex regulatory node (Layer 1), pathway modules as intermediate nodes (Layer 2), and individual molecular targets as terminal gene/protein nodes (Layer 3). Cross-module edges were drawn between nodes with established biological interactions documented in the literature, including nuclear factor erythroid 2-related factor 2 (NRF2)-mediated regulation of mitochondrial biogenesis and senescence, histone deacetylase (HDAC)/DNA methyl transferase (DNMT) epigenetic control of antioxidant transcription, p53-mitochondrial integrity signaling, and mitochondrial dysfunction-driven p16^INK4a^ (p16) induction. Node border color encodes regulation direction relative to WT-SED: a red border indicates upregulation (FC > 1.10), blue indicates downregulation (FC < 0.90), and grey indicates no substantial change (FC 0.90–1.10). The network was constructed and visualized in Python v3.10.14 using the Matplotlib library v3.x [35].

## 3. Results

### 3.1. Physical Exercise Activation of Antioxidant and Detoxifying Genes in the 5XFAD Mouse Brain

**Figure 1:**
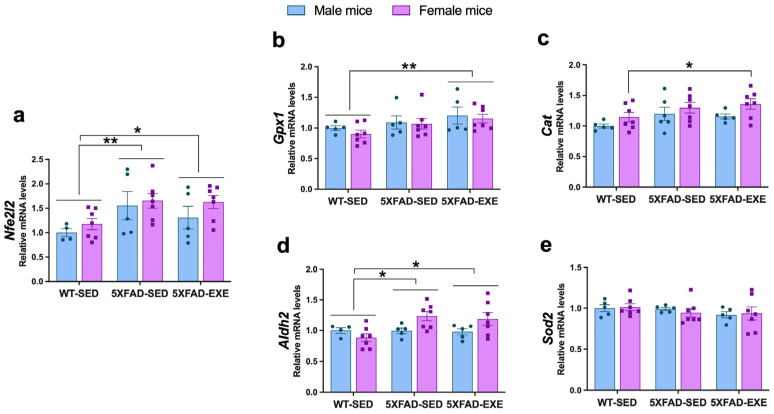
Expression levels of antioxidant defense genes in the cerebral cortices of male and female 5XFAD mice subjected to voluntary wheel running. (**a**) *Nfe2l2*. (**b**) *Gpx1*. (**c**) *Cat*. (**d**) *Aldh2*. (**e**) *Sod2*. Absence of sex effect and further analysis of pooled male and female data in (**a**,**b**,**d**). Values are presented as mean ± SEM (Male mice: WT-SED *N* = 5, with one outlier excluded from the *Nfe2l2* and *Aldh2* datasets (final *N* = 4); 5XFAD-SED *N* = 5; 5xFAD-EXE *N* = 5. Female mice: WT-SED *N* = 7; 5XFAD-SED *N* = 7; 5XFAD-EXE *N* = 7). Statistics: * *p* < 0.05, ** *p* < 0.01.

Gene expression of the key transcription factor nuclear factor erythroid 2-like 2 (NFE2L2; also known as NRF2), the first-line antioxidant enzymes glutathione peroxidase 1 (GPX1) and catalase, and mitochondrial aldehyde dehydrogenase 2 (ALDH2) and superoxide dismutase 2 (SOD2) were determined in the cerebral cortex (Figure 1a–e). Two-way ANOVA showed a sex factor effect (*p* = 0.031) and a group factor effect (*p* = 0.041) in *Cat* expression. There was no sex significance for the other genes, and the male and female data were pooled for analysis by one-way ANOVA. Physical exercise induced increases *Gpx1* mRNA expression in male and female 5XFAD-EXE mice and *Cat* mRNA expression in female mice. There were significant increases in *Nfe2l2* and the mitochondrial aldehyde detoxifier *Aldh2* in male and female mice in the 5XFAD-SED and 5XFAD-EXE groups. No changes were detected in the mitochondrial antioxidant gene *Sod2*. This indicates a preserved mitochondrial status. However, the general activation of the cell antioxidant genes would improve cell defense against free radicals and oxidized molecules in exercised mice.

### 3.2. Physical Exercise Boosts Mitochondrial Subunit Dynamics in the 5XFAD Mouse Brain

**Figure 2:**
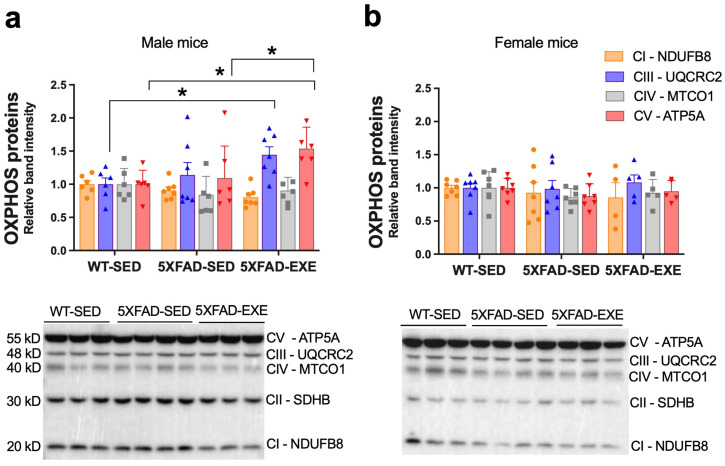
Protein levels of essential subunits of oxidative phosphorylation complexes (OXPHOS) in the hippocampi of 5XFAD mice subjected to voluntary wheel running. (**a**) Densitometric western blot analysis and cropped representative blots from male mice. (**b**) Densitometric analysis and cropped representative blots from female mice. The values of the subunits of mitochondrial complexes I, III, IV, and V were normalized to the respective complex II subunit value. Full-length western blot images are shown in Supplementary Figure S1. Values are presented as mean ± SEM (Male mice: WT-SED *N* = 6; 5XFAD-SED *N* = 7; 5XFAD-EXE *N* = 7. Female mice: WT-SED *N* = 7; 5XFAD-SED *N* = 7; 5XFAD-EXE *N* = 5, with one outlier excluded from the CI—NDUFB8 dataset (final *N* = 4)). Statistics: * *p* < 0.01.

Analysis of the mitochondrial enzymatic complexes in the hippocampal mouse tissue showed preservation of the proteins constituting the mitochondrial electron transport chain in 5XFAD mice and modulation by physical exercise (Figure 2a,b). Two-way ANOVA showed effects of the mitochondrial complex factor (*p* < 0.001), the group factor (*p* = 0.047), and their interaction (*p* = 0.018) in males, and no effects in females. Relative levels of the complex protein markers were similar between 5XFAD-SED mice and their sibling WT-SED mice. Notably, 5XFAD-EXE males, but not females, showed a significant increase of relative levels of CIII and CV proteins. Therefore, males subjected to voluntary aerobic exercise showed increased levels of key proteins involved in mitochondrial oxidative phosphorylation.

### 3.3. Physical Exercise Modulation of Epigenetic Genes in the 5XFAD Mouse Brain

**Figure 3:**
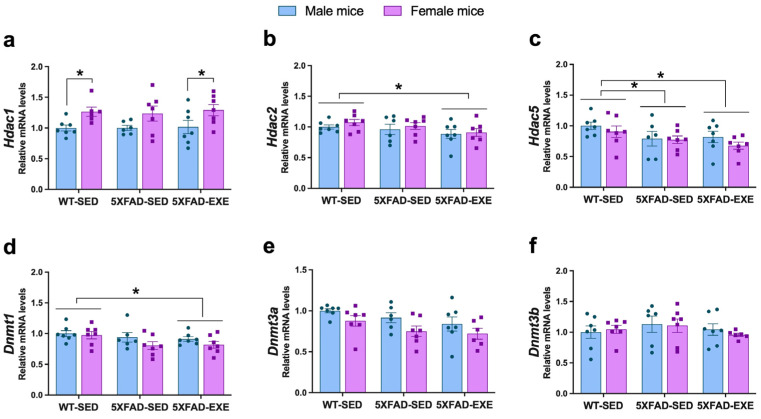
Expression levels of genes involved in epigenetic processes in the hippocampi of male and female 5XFAD mice subjected to voluntary wheel running. (**a**) *Hdac1*. (**b**) *Hdac2*. (**c**) *Hdac5*. (**d**) *Dnmt1*. (**e**) *Dnmt3a*. (**f**) *Dnmt3b*. Absence of sex effect and further analysis of pooled male and female data in (**b**–**f**). Values are presented as mean ± SEM (Male mice: WT-SED *N* = 7; 5XFAD-SED *N* = 6; 5XFAD-EXE *N* = 7. Female mice: WT-SED *N* = 7, with one outlier excluded from the *Hdac1* and *Hdac2* datasets (final *N* = 6); 5XFAD-SED *N* = 7; 5XFAD-EXE *N* = 7, with one outlier excluded from the *Dnmt3a* dataset (final *N* = 6)). Statistics: * *p* < 0.05.

Changes in the mRNA levels of genes involved in epigenetic processes were analyzed in hippocampal tissue from male and female 5XFAD mice and WT sibling mice. Expression of the HDAC enzyme genes *Hdac1*, *Hdac2*, and *Hdac5* and the DNMT enzyme genes *Dnmt1*, *Dnmt3a,* and *Dnmt3b* were examined in the WT-SED, 5XFAD-SED, and 5XFAD-EXE experimental groups (Figure 3a–f). In a two-way ANOVA, the sex factor effect reached significance only for *Hdac1* (*p* = 0.001) and *Dnmt3a* (*p* = 0.014), which showed higher and lower mRNA levels in females, respectively. In addition, there was a group factor effect (*p* = 0.050) for *Dnmt3a*, but not for *Hdac1*. The results for the other genes were analyzed using pooled data from males and females. Expression of *Hdac2* showed a decrease in 5XFAD-EXE mice compared to WT-SED mice, while expression of *Hdac5* and *Dnmt1* showed a decrease in 5XFAD-SED mice and 5XFAD-EXE mice compared to WT-SED mice. No changes were detected in *Dnmt3b*. Therefore, the significant epigenic modulation induced by physical exercise may be related to long-term improved neural function.

### 3.4. Physical Exercise Improves Proteasomal Function in the 5XFAD Mouse Brain and the Whole Blood of Middle-Aged Amateur Rugby Players

**Figure 4:**
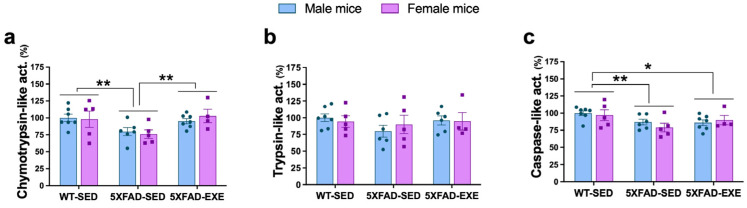
Hydrolytic activity of the proteasome in the cerebral cortices of male and female 5XFAD mice subjected to voluntary wheel running. (**a**) Chymotrypsin-like activity. (**b**) Trypsin-like activity. (**c**) Caspase-like activity. Values are presented as mean ± SEM (Male mice: WT-SED *N* = 7; 5XFAD-SED *N* = 6; 5XFAD-EXE *N* = 7, with one outlier excluded from the trypsin-like activity dataset (final *N* = 6). Female mice: WT-SED *N* = 5; 5XFAD-SED *N* = 5; 5XFAD-EXE *N* = 4). Absence of sex effect and further analysis of pooled male and female data in (**a**–**c**). Statistics: * *p* < 0.05, ** *p* < 0.01.

Activation of proteasome enzymatic hydrolysis of aberrant proteins by physical exercise was analyzed in mouse cerebral cortical tissue (Figure 4a–c). The results showed no sex factor effects, and data for males and females were pooled for further statistical analysis. The specific chymotrypsin- and caspase-like activities showed statistically significant decreases in the 5XFAD group. Chymotrypsin-like activity was totally restored in the 5XFAD-EXE group. The recovery of caspase-like activity was not significant. No effects were detected on trypsin-like activity for any of the experimental groups. The catalytic sites responsible for the chymotrypsin-like, trypsin-like and caspase-like activities are located in the proteasomal subunits β5, β2, and β1, respectively. Alternatively, subunits β5i, β2i, and β1i of the immunoproteasome can also contribute to these respective activities.

**Figure 5:**
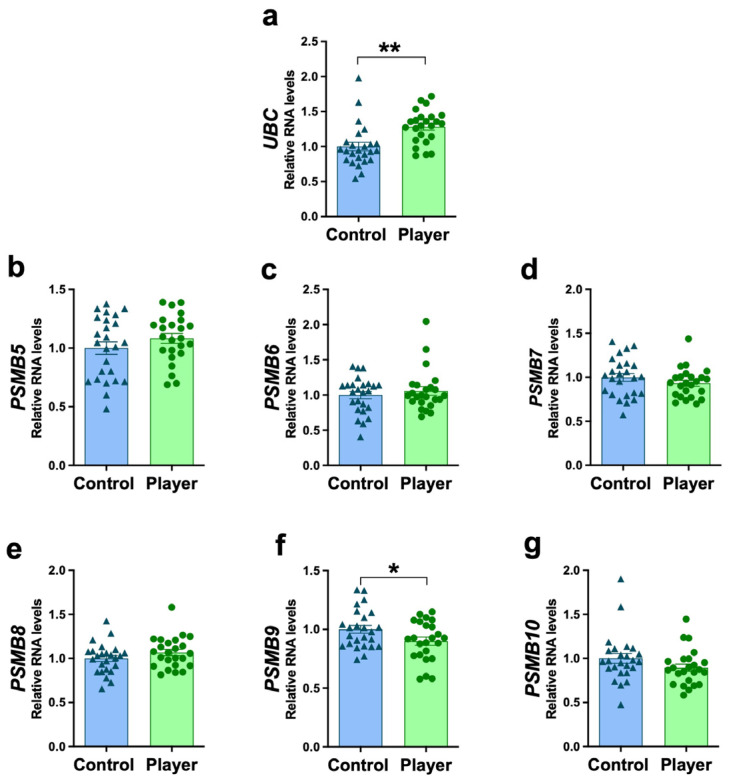
Expression levels of genes encoding components of the ubiquitin–proteasome system in whole blood from middle-aged male veteran rugby players (Player) and age-matched men with low physical activity (Control). (**a**) *UBC*. (**b**) *PSMB5*. (**c**) *PSMB6*. (**d**) *PSMB7*. (**e**) *PSMB8*. (**f**) *PSMB9*. (**g**) *PSMB10*. Values are presented as mean ± SEM (Control group *N* = 25; Player group *N* = 24). Statistics: * *p* < 0.05, ** *p* < 0.001.

Modulation of proteasome dynamics by physical exercise was analyzed in human blood samples. The expression of selected genes, including ubiquitin C and catalytic subunits of the constitutive proteasome and the immunoproteasome, was determined in whole blood from middle-aged male veteran rugby players and sedentary controls. mRNA levels of the polyubiquitin precursor *UBC* were increased in the blood samples from rugby players compared to the control group (Figure 5a). The genes *PSMB5*, *PSMP6*, and *PSMB7,* encoding the subunits *β*5, *β*1, and *β*2 of the constitutive proteasome, respectively, showed similar mRNA levels (Figure 5b–d). Among the immunoproteasome subunits, there was a decrease in mRNA expression of *PSMB9*, which encodes *β*1i, and no changes in the mRNA levels of *PSMB8* and *PSMB10*, which encode the *β*5i and *β*2i subunits, respectively (Figure 5e–g). Activation of ubiquitin and a decrease in the less efficient immunoproteasome components indicate improved proteasomal function.

### 3.5. Physical Exercise Reduces Senescence Markers in the 5XFAD Mouse Brain and the Whole Blood of Middle-Aged Amateur Rugby Players

**Figure 6:**
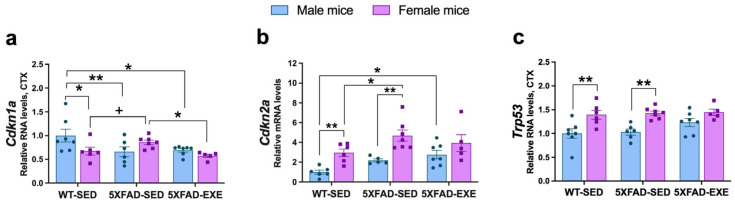
Expression levels of senescence genes in the cerebral cortex of male and female 5XFAD mice subjected to voluntary wheel running. (**a**) *Cdkn1a*. (**b**) *Cdkn2a*. (**c**) *Trp53*. Values are presented as mean ± SEM (Male mice: WT-SED *N* = 7, with one outlier excluded from *Cdkn2a* (final *N* = 6); 5XFAD-SED *N* = 6, with one outlier excluded from *Cdkn2a* dataset (final *N* = 5); 5XFAD-EXE *N* = 7. Female mice: WT-SED *N* = 6; 5XFAD-SED *N* = 7; 5XFAD-EXE *N* = 5). Statistics: * *p* < 0.05, ** *p* < 0.01, + *p* = 0.06 (borderline significance).

Changes in the mRNA levels of genes involved in the main senescence pathways were analyzed in mouse cerebral cortical tissue and human whole blood. The mouse genes *Cdkn1a*, *Cdkn2a*, and *Trp53* encode the cyclin-dependent kinase inhibitor 1A or p21, the cyclin-dependent kinase inhibitor 2A or p16, and the tumor suppressor protein p53, respectively. They all induce cell cycle arrest and senescence. Their expression levels showed group differences in modulation by physical exercise (Figure 6a–c). In two-way ANOVA, there was an interaction between the factors sex and group in the mRNA levels of *Cdkn1a* (*p* = 0.015), a sex effect in those of *Cdkn2a* (*p* < 0.001) and *Trp53* (*p* <0.001), and a group effect in *Cdkn2a* mRNA levels (*p* = 0.008). The group mean analysis showed higher expression levels of *Cdkn2a* and *Trp53* in females than in males for the sedentary groups WT-SED and 5XFAD-SED, but no sex differences were found in the 5XFAD-EXE mice. Furthermore, female 5XFAD mice showed an increase in *Cdkn1a* and *Cdkn2a* levels compared to female WT-SED mice that was no longer significant in the exercised females (5XFAD-EXE). Furthermore, the expression decrease was significant for *Cdkn1a*. However, the male groups showed an irregular pattern, without consistent effects of the gene, AD model, or exercise intervention. Therefore, 5XFAD-SED females showed activation of senescence pathways involving the genes *Cdkn2a1* (p21) and *Cdkn2a* (p16) that was inhibited by aerobic physical exercise.

**Figure 7:**
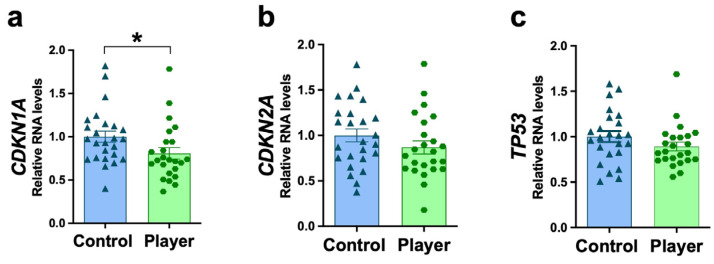
Expression levels of senescence genes in whole blood from middle-aged male veteran rugby players (Player) and age-matched men with low physical activity (Control). (**a**) *CDNK1A*. (**b**) *CDKN2A*. (**c**) *TP53*. Values are presented as mean ± SEM (Control group *N* = 25, with one outlier excluded from the *TP53* dataset (final *N* = 24); Player group *N* = 24). Statistics: * *p* < 0.05.

Long-term physical exercise in veteran rugby players also modulated the expression of genes associated with senescence pathways (Figure 7a–c). Leisure players showed decreased levels of the senescence gene *CDKN1A* compared to controls with low physical activity. No statistically significant changes were detected in the whole-blood mRNA levels of *CDKN2A* and *TPR5*, despite a general decreasing trend.

### 3.6. Network Analysis of 5XFAD Genes and Proteins Reveals Coordinated Pathway Modules Underlying Exercise-Induced Neuroprotection

**Figure 8:**
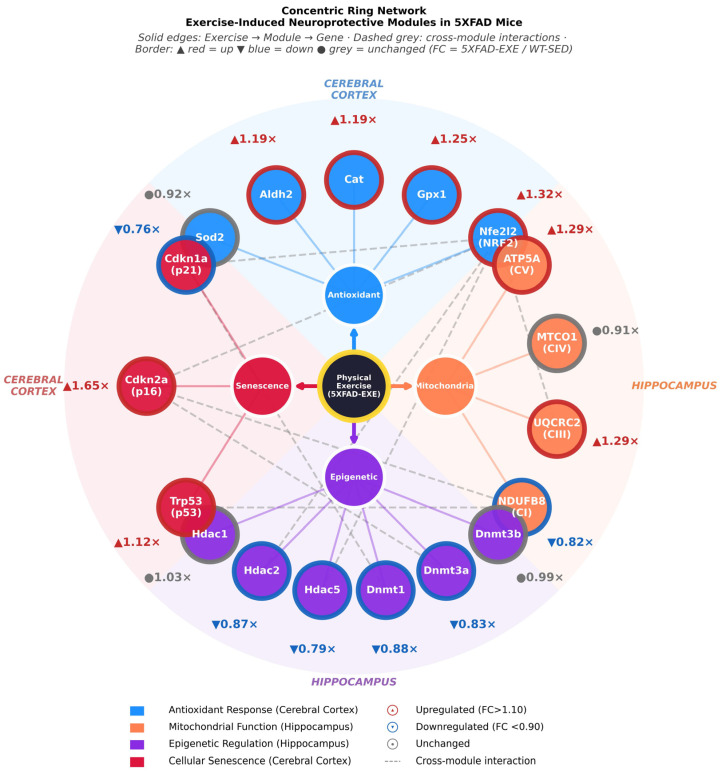
Hierarchical gene/protein regulatory network of exercise-induced neuroprotective modules in 5XFAD mice. The network is organized in three layers: physical exercise (central node, master regulatory signal), four biological pathway modules (inner ring), and individual gene/protein targets (outer ring). Solid colored edges represent Exercise → Module → Gene directed relationships; grey dashed edges indicate established cross-module biological interactions (NRF2–senescence/mitochondrial axis, HDAC/DNMT epigenetic control of antioxidant transcription, p53–mitochondrial integrity crosstalk). Node colors indicate pathway module: blue = antioxidant response (cortex); orange = mitochondrial function (hippocampus); purple = epigenetic regulation (hippocampus); red = cellular senescence (cortex). Node border color encodes regulation direction in 5XFAD-EXE vs. WT-SED: red border ▲ = upregulated (FC > 1.10); blue border ▼ = downregulated (FC < 0.90); grey border ● = unchanged (FC 0.90–1.10). Values adjacent to each gene node indicate mean fold change (5XFAD-EXE/WT-SED). FC, fold change; WT-SED, sedentary wild type; 5XFAD-EXE, exercised 5XFAD transgenic mice.

To step beyond individual gene expression changes and understand the system-wide organization of the exercise response, all molecular targets from Figure 1, Figure 2, Figure 3 and Figure 6 were combined into a hierarchical gene/protein regulatory network (Figure 8). This network grouped the 18 molecular targets into four biologically meaningful pathway modules, all converging on physical exercise as the main regulatory signal. The antioxidant response module (cerebral cortex) was the most strongly upregulated, with *Nfe2l2*, *Gpx1*, *Cat*, and *Aldh2* showing FC > 1.10 in 5XFAD-EXE mice, while *Sod2* remained stable (FC = 0.92), indicating maintained mitochondrial redox balance. The mitochondrial function module (hippocampus) exhibited selective upregulation of UQCRC2/CIII (FC = 1.29) and ATP5A/CV (FC = 1.29), highlighting exercise-related enhancement at specific electron transport chain steps. The epigenetic regulation module (hippocampus) was mainly downregulated, with *Hdac2*, *Hdac5*, *Dnmt1*, and *Dnmt3a* all decreased (FC = 0.79–0.88), while *Hdac1* and *Dnmt3b* showed no change. The cellular senescence module (cerebral cortex) displayed contrasting patterns: *Cdkn1a*/p21 was downregulated (FC = 0.76), whereas *Cdkn2a*/p16 was upregulated (FC = 1.65). Cross-module interactions reveal important functional links, such as NRF2-mediated mitochondrial and anti-senescence pathways, epigenetic repression of antioxidant genes, and crosstalk between *Trp53*/p53 and mitochondrial integrity.

### 3.7. Physical Exercise Protection Against Behavioral Changes and Memory Loss in 5XFAD Mice

Behavioral analysis to detect BPSD-like symptoms and memory changes was performed in male 5XFAD mice and their male WT littermates. A timeline of non-treated mice included outcomes at the age when the exercise treatment would begin, at an intermediate time point, and at the termination point (Supplementary Figure S2). Young adult (2-month-old) and middle-aged (4-month-old) 5XFAD mice suffered memory loss but not BPSD-like changes compared to age-matched WT mice. However, at the advanced age of 8 months for this strain, 5XFAD mice suffered both memory loss and BPSD-like pathologies. These mice showed reduced movement and exploratory activity in the open field arena, including both ambulation and rearings, greater anxiety to enter or remain in the lit compartment of the light–dark apparatus, and higher apathy or lack of curiosity reflected by a decreased number of head dips in the four-hole-board test. Meanwhile, poor memory response in the NORT was evident from the earliest age tested, as mice poorly discriminate a novel object from a familiar object previously explored.

**Figure 9:**
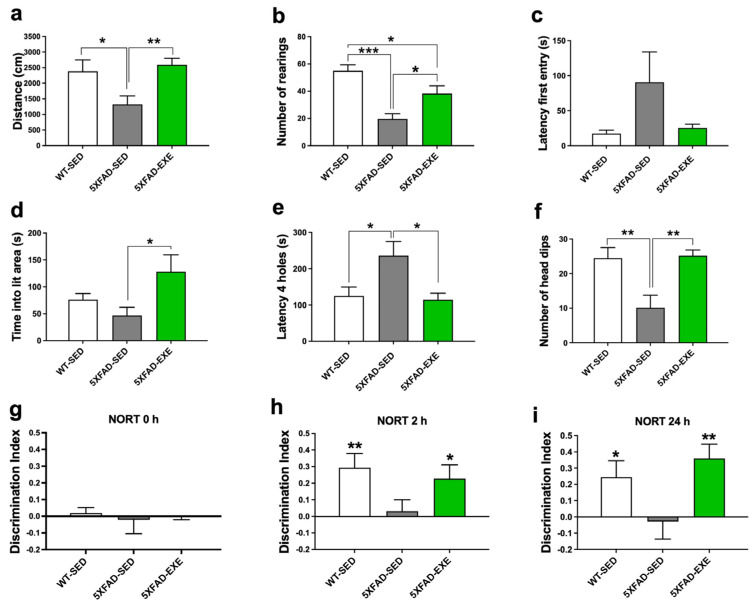
Preservation of general and cognitive behavior in male 5XFAD mice subjected to voluntary wheel running. (**a**,**b**) Horizontal and vertical activity in the open field test. (**c**,**d**) Latency to entry and time into the lit area in the light–dark test. (**e**,**f**) Latency to explore the four holes and total number of head dips in the four-hole-board test. (**g**–**i**) Acquisition, short-term memory, and long-term memory in the novel object recognition test (NORT). Values are presented as mean ± SEM (*N* = 6 per group, with one outlier excluded from the 5XFAD-EXE group in the light–dark test (final *N* = 5)). Statistics: * *p* < 0.05, ** *p* < 0.01, *** *p* < 0.001.

Therefore, BPSD-like behavior and memory changes were analyzed in 8-month-old 5XFAD-EXE mice subjected to 6 months of voluntary wheel running to characterize their brain functionality in comparison to 5XFAD-SED and WT-SED mice (Figure 9). Older 5XFAD-EXE mice showed an absence of BPSD-like changes and preservation of memory. Notably, they exhibited increased horizontal and vertical exploratory activity in the open field test, reaching or equaling WT-SED levels (Figure 9a,b). In the light–dark box, 5XFAD-EXE mice entered the lit compartment earlier, as indicated by reduced latency, and spent significantly more time there, reflecting lower anxiety than 5XFAD-SED mice (Figure 9c,d). Similarly, 5XFAD-EXE mice showed the same level of curiosity as WT-SED mice in the latency of exploration of all four holes and in total hole explorations in the four-hole-board test (Figure 9e,f). Furthermore, in the NORT, they showed a null discrimination index of two identical objects (time 0 h) but a significant discrimination index both at both short-term (2 h) and long-term (24 h) delays after exploring one familiar object and a novel object (Figure 9g–i). The behavioral results of exercised 5XFAD mice showed preserved emotional responses and short-term and long-term memory.

## 4. Discussion

Long-term physical exercise induced neuroprotective changes in 5XFAD brain function that paralleled molecular and cellular adaptations. The results agree with the proposal that redox signaling is an early event in the multiple protective pathways activated by physical exercise [36].

The antioxidant response is a well-established downstream pathway. Accordingly, 8-month-old 5XFAD mice subjected to voluntary wheel running for 6 months showed increased expression of first-line antioxidant and detoxifying genes in the cerebral cortex. The expression of the gene *Nfe2l2,* which encodes the transcription factor NFE2L2/NFR2, was upregulated in both 5XFAD groups. NRF2 is a master regulator of the response to the oxidative stress generated by physical exercise and other stressors. In response to exercise, NRF2 activates multiple downstream antioxidants and detoxifying pathways, including mitochondrial remodeling, and creates a hormetic response against future damage [37,38]. It has been proposed as a therapeutic target in AD [39]. Increased NRF2 protein levels have been reported in the hippocampi of APP/PS1 mice subjected to swimming [40]. Human *NFE2L2* mRNA has also been shown to be increased in the blood of middle-aged male rugby players included in the current study [30]. In addition to its activation in exercised mice, the increased *Nfe2l2* levels in sedentary 5XFAD might orchestrate a defense against oxidative damage and aberrant proteins in the AD-like brain.

Furthermore, the increase in *Gpx1* is highly relevant, as the encoded enzyme GPX1 detoxifies hydroperoxides throughout cells. Similarly, the cerebral cortices of male and female 3xTg-AD mice subjected to the same exercise treatment have shown increased GPX1 activity, together with elevated levels of glutathione (GSH) [34]. Catalase, another ubiquitous enzyme that decomposes hydrogen peroxide, showed increased *Cat* mRNA only in exercised 5XFAD females, indicating a higher adaptive antioxidant response to exercise in females. However, catalase gene expression has previously been shown to increase in the cerebral cortices of male WT mice exposed to forced running [41] and in the peripheral blood of our cohort of middle-aged male rugby players [30]. Catalase and GSH protein levels have also been shown to be increased in serum from patients with Parkinson’s disease following an intervention of aerobic exercise [42].

Similarly, the mitochondrial ALDH2 gene *Aldh2* was upregulated in both 5XFAD groups, suggesting activation of antioxidant defenses against toxic aldehydes. This aligns with the reported reduced lipid peroxidation-derived malondialdehyde in peripheral plasma from young and middle-aged exercise-trained men [29]. Enhancement of ALDH2 activity has been proposed as a strategy against AD [43]. In contrast, the mitochondrial SOD2 gene *Sod2* did not show activation, indicating the absence of oxidative phosphorylation dysfunction that would increase superoxide anion generation. Non-strenuous physical exercise is proposed to be a mild stressor that improves the antioxidant response in the brain [41]. Antioxidant enhancement has been proposed as a central neuroprotective response in aging and AD, conditions characterized by oxidative stress [44].

Maintained protein levels of mitochondrial complex proteins in sedentary 5XFAD mice suggests a functional oxidative phosphorylation machinery in these mice. Interestingly, a relative increase in CIII and CV protein levels was induced by physical exercise in male 5XFAD mice, but not in female 5XFAD mice. Female rodents have been reported to be less responsive than males to exercise-induced enhancement of mitochondrial electron transfer activity [45,46]. Increased activities of CI, CIII, or CIV have been reported in rodent brains after exercise training [45,47,48]. The ubiquinol–cytochrome c reductase CIII complex is a central component essential for continuity of electron transport. ATP synthase CV mediates the final step of oxidative phosphorylation and is essential for sustaining the high energy demands of neurons. Therefore, the results with exercised 5XFAD mice suggest a potential enhancement of brain mitochondrial activity and ATP generation in males, although further confirmation is needed. Consistently, previous results from the middle-aged human cohort included in this study demonstrated that physical activity restores the expression of the key mitochondrial regulator *SIRT3* to youthful levels [30].

Epigenetic dynamics are another downstream pathway of redox signaling initiated by physical exercise [49]. Key HDAC (*Hdac2*, *Hdac5*) and DNMT (*Dnmt1* and *Dnmt3a*) epigenetic genes showed lower hippocampal expression in exercised 5XFAD mice compared to WT mice. However, *Hdac5* mRNA levels were also decreased in sedentary 5XFAD mice. HDAC2 activity is a negative regulator of hippocampus-dependent learning and memory that reduces the transcription of synaptic plasticity genes [50]. HDAC5 is involved in the negative modulation of cognitive and behavioral processes, such as object recognition memory and sociability in mice [51]. Similar to our results, reduced mRNA levels of *Hdac2* have been reported in the hippocampus of WT mice subjected to 30 days of voluntary running exercise [52], and reduced *Hdacs5* levels after 1 week of exercise [53]. Epigenetic regulation of plasticity factors such as BDNF through histone acetylation may contribute to the cognitive effects induced by exercise [54]. Inhibitors of HDAC activity have been proposed as cognitive enhancers and therapeutic tools against cognitive impairment [55]. DNMTs play a complex role in the dynamic modulation of DNA methylation of specific genes, including those involved in neuroplasticity and repair. Pro-cognitive interventions such as physical exercise in young rodents have shown reductions in DNMT expression [53], consistent with our findings in 5XFAD mice. These authors reported that 1 week of running exercise in WT mice decreases mRNA levels of *Dnmt1* and *Dnmt3a*, and DNMT gene expression negatively correlates with BDNF and synaptic plasticity markers.

Therefore, epigenomic changes induced by physical exercise are known to activate neuroplasticity factors that improve brain function and cognition [49]. Epigenetic changes are investigated as potential biomarkers of AD prognosis and therapeutic strategies [56], in line with the consistent findings in AD mouse models.

Proteasomal function is activated by redox signaling through NRF2 [57]. Therefore, long-term voluntary physical exercise was expected to improve the ubiquitin–proteasome system in the mouse brain and human blood cells [58]. Accordingly, voluntary wheel running in 5XFAD mice induced recovery of chymotrypsin-like activity, the major hydrolytic activity of the proteasome. This would improve the elimination of abnormal proteins linked to the amyloid burden in these mice. Likewise, a pharmacological treatment that increases protein levels of the proteasome catalytic subunit *β*5, which is responsible for chymotrypsin-like activity, has been shown to reduce amyloid burden and ameliorate behavioral phenotypes in 5XFAD mice [59]. In the AD brain, there is an accumulation of non-degraded ubiquitinated proteins due to the impaired proteasomal hydrolytic activity [60]. Amyloid oligomers have been shown to inhibit chymotrypsin-like and caspase-like proteasome activities in human brain slices [61].

Modulation of proteasome activity is largely post-translational. Therefore, no changes were detected in mRNA levels of the constitutive proteasome units in blood from rugby players. However, *UBC*, which encodes ubiquitin, was increased by physical exercise, suggesting upregulation of this gene as a response to exercise-induced stress, as described for other stress inducers [62]. This represents a hormetic response with long-term benefits. Furthermore, the decrease in *PSMB9*, which encodes a subunit of the immunoproteasome, indicates better preservation of the constitutive proteasome, higher functionality, and an anti-aging effect in the exercised group. These translational results support the potential of physical exercise interventions to reduce amyloid aggregates and eliminate damaged proteins.

The molecular and physiological improvements induced by aerobic exercise in the mouse brain and human blood cells should have impacted their senescence status. Accordingly, screening of cell cycle genes as markers of senescence showed significant decreases in the exercised female 5XFAD mouse brain and in blood cells from amateur rugby players. Specifically, senescence markers in 5XFAD mice indicated greater relevance of *Cdkn2a* (encoding p16) than *Cdkn1a* (encoding p21) or *Trp53*, consistent with previous reports [63]. Notably, exercised females displayed lower p21 and p16 gene expression than their sedentary counterparts, fully or partially reverting to WT levels, respectively. Increased expression of cyclin-dependent kinases and unexpected expression of their inhibitors have long been interpreted as aberrant cell cycle re-entry in terminally differentiated neurons in AD. For instance, cyclin-dependent kinase 4 and its inhibitor p16 have been immunolabeled in AD brain tissue [64]. A p16 increase has been reported in peripheral mononuclear cells of AD patients [65]. The 5XFAD results suggest that physical exercise reduces cellular senescence induced by AD neuropathology. However, further analysis of senescence phenotype markers would be required to confirm the biological relevance of the gene expression changes observed in this study. Previous studies on exercise’s anti-senescence effects on brain cells are scarce. Some protective effects have been described in the microglia of aged mice [66] or brain tissue from mice with metabolically induced senescence [67].

In the sportsmen cohort, the decrease in *CDKN1A* (encoding p21) in peripheral blood cells from middle-aged rugby players may be linked to the lower level of circulating inflammatory markers and higher pro-youthful factors previously reported in this cohort [30]. These results agree with the reported decrease in circulating senescence biomarkers in older men participating in an exercise intervention [68]. Given the blood–brain communication described in several experimental settings and anti-aging interventions, a shift from pro-aging to pro-youthful factors in peripheral blood likely indicates anti-aging effects on the brain [69]. Therefore, these data provide potential translational support for the anti-senescence effects of exercise.

Integrating mouse multi-omics data across brain regions into a hierarchical network (Figure 8) showed that gene and protein changes in 5XFAD-EXE mice do not occur in isolation, but form four interconnected pathway modules, all centered around physical exercise as the main regulatory trigger. The network highlights the NRF2-driven antioxidant response module as a hub that connects mitochondrial function, epigenetic regulation, and cellular senescence, aligning with NRF2’s known role as a versatile regulator that links redox signaling to bioenergetics, chromatin remodeling, and cell cycle control. The concurrent downregulation of HDAC and DNMT genes in the epigenetic regulation module, which is linked to the antioxidant transcriptional program, indicates that exercise-induced epigenomic relaxation promotes sustained activation of NRF2 target genes. The inverse pattern of *Cdkn1a*/p21 downregulation and *Cdkn2a*/p16 upregulation within the senescence module, along with their connections to mitochondrial markers, suggests a complex reorganization of cell cycle arrest pathways rather than a straightforward anti-senescence process. Overall, this network analysis reveals that the neuroprotective effects of long-term aerobic exercise in the 5XFAD brain stem from the coordinated regulation of these interconnected modules, supporting the idea that redox signaling triggered by muscle contraction acts as a master upstream signal, simultaneously activating antioxidant defenses, bioenergetic adaptation, epigenomic flexibility, and senescence control in the aging AD brain.

Mouse proteasome activity and human proteomics and senescence-related omics data could not be integrated into this network due to differences in data categories. However, proteasomal activity plays a well-established role in counteracting cellular senescence, given its essential function in maintaining cell survival [70]. Therefore, we speculate that the reported effects on these systems may also be, at least partially, modulated by NRF2-activated genes. A more in-depth analysis of NRF2 redox signaling will be required to confirm its central role in exercise-induced adaptive mechanisms. Furthermore, the cellular pathways discussed here may contribute to enhancing brain defenses against aging and AD-related neurodegeneration, in agreement with previous reports on the beneficial effects of physical exercise on antioxidant capacity, epigenetic regulation, and mitochondrial fitness [71,72,73].

We observed some sex-dependent responses throughout the study, with the higher senescence status in females being the most striking. Compared to males, female 5XFAD mice showed elevated senescence gene expression, whereas senescence-associated genes encoding p21 and p16 were markedly decreased by exercise. Higher levels of senescence biomarkers may relate to the greater amyloid burden reported in females compared to males [26,74]. Nevertheless, no differences in longevity have been described between male and female 5XFAD mice [75]. Furthermore, we may speculate that hormonal factors contribute to the differential antioxidant and mitochondrial sex responses to the physical exercise treatment. Estrogen signaling interacts with NRF2 activity and downstream antioxidant gene expression [76] and may underly the higher response observed in some antioxidant enzymes, such as catalase. Conversely, the male-specific increase in mitochondrial protein levels of CIII and CV, crucial bioenergetic units, may reflect a testosterone-driven enhancement of mitochondrial gene expression [77] concomitant with the exercise effect. This male hormone protects brain mitochondria from aging and disease [78]. Further benefits of female hormones relate to epigenetic modulation through estrogen-responsive elements that may have mediated the minor sex-related changes found in basal epigenetic markers [79]. Future research should explore these hypotheses through in-depth, sex-specific profiling of exercise effects, as well as co-treatment with selective estrogen or testosterone receptor modulators in AD models.

Finally, the beneficial effects of exercise on brain function were confirmed by emotional behavior and memory testing in male 5XFAD mice. Loss of recognition memory has previously been reported in male and female 5XFAD mice at the moderate pathological stage of 4 months [80,81] and at more advanced stages [82]. However, we observed memory deficits already at the onset of treatment, at 2 months of age. At this stage, the mice were in an early phase of AD-like pathology, with initial amyloid deposition and mild gliosis [26]. Despite these early cognitive impairments, BPSD-like features developed later. Reduced activity and exploration, anxiety, and apathy were observed in 5XFAD mice at 8 months of age. Remarkably, free access to a running wheel from 2 to 8 months of age completely protected the mice from cognitive decline and BPSD-like changes. This demonstrates that the signaling pathways and downstream brain changes induced by physical exercise as discussed above protected 5XFAD mice against AD-like dementia.

Consistent with the overlap in pathways identified in the human cohort, physically active participants showed better memory performance in free and cued selective reminding tests than low-activity controls [29]. Therefore, exercise-induced pathways appeared to protect against age-related memory decline in middle age. The 6-month period of voluntary running in the mice is approximately equivalent to 20 years of human life [28]. This timeframe coincides with the training years of the cohort of rugby players analyzed here, further reinforcing the potential of the translational findings regarding fitness and anti-aging effects. Future studies should define the optimal exercise parameters and investigate how these adaptive pathways can be leveraged for preventive or therapeutic strategies in human aging and neurodegeneration.

Limitations of the study include potential bias and imprecision associated with the experimental design in 5XFAD mice, as well as the use of peripheral blood from humans. The mouse study focused primarily on protective effects against AD-like neurodegeneration, but the absence of exercised WT mouse group reduced the strength of the conclusions. In addition, the absence of behavioral and cognitive testing in females prevented the linking of some sex-related molecular effects to potential quantitatively differential neuroprotection between sexes. Furthermore, the lack of recording of wheel running activity limited the finding of further associations between exercise performance and study outcomes. Finally, although peripheral whole blood is increasingly used as a surrogate for brain biomarker testing, their changes in the blood may not fully reflect central nervous system processes.

## 5. Conclusions

Long-term voluntary physical exercise exerts comprehensive neuroprotective effects in 5XFAD mice, a recognized model of familial AD. Molecular analysis of brain tissue showed gene expression and protein changes associated with enhanced antioxidant defense and mitochondrial function, decreased senescence, and improved epigenetic regulation. These adaptations were associated with preserved memory and general behavior. Multi-omics network analysis revealed four interconnected pathway modules—antioxidant response, mitochondrial function, epigenetic regulation, and cellular senescence—driven by physical exercise, with NRF2 acting as a central hub linking redox signaling to energy production, chromatin remodeling, and senescence regulation.

Similar improvements in proteasome- and senescence-related gene expression in blood cells from long-term amateur rugby players potentially support the translational relevance of these findings.

These results underscore the therapeutic promise of exercise interventions in age-related cognitive decline and AD and identify the NRF2–epigenetic–mitochondrial pathway as a promising target for exercise-mimetic drugs. Further studies are warranted to validate the omics findings at the cellular phenotype and functional levels and to clarify sex-specific responses in order to develop tailored interventions.

## Data Availability

The original contributions presented in this study are included in the article/Supplementary Material. Further inquiries can be directed to the corresponding authors.

## Supplementary Materials

The following supporting information can be downloaded at: https://www.mdpi.com/article/10.3390/antiox15060698/s1, Table S1: List of target genes and their corresponding TaqMan FAM-labeled probes for real-time qPCR. Figure S1: Full western blot membranes probed with anti-OXPHOS antibodies. Figure S2: Time course of cognition and general behavior in 5XFAD and WT male mice.

## Abbreviations

The following abbreviations are used in this manuscript:

5XFAD-EXE	Exercised 5XFAD Mice
5XFAD-SED	Sedentary 5XFAD Mice
AD	Alzheimer’s Disease
ALDH2	Aldehyde Dehydrogenase 2
ATP5A	CV—ATP Synthase Subunit 5
BDNF	Brain-Derived Neurotrophic Factor
BPSD	Behavioral and Psychological Symptoms of Dementia
cDNA	First-Strand Complementary DNA
DNMT	DNA Methyl Transferase
FC	Fold Change
GPX1	Glutathione Peroxidase 1
GSH	Glutathione
HDAC	Histone Deacetylase
MTCO1	CIV—Cytochrome C Oxidase Subunit 1
NDUFB8	CI—NADH:Ubiquinone Oxidoreductase Subunit B8
NRF2/NFE2L2	Nuclear Factor Erythroid 2-Related Factor 2/Nuclear Factor Erythroid 2-Like 2
NORT	Novel Object Recognition Test
OXPHOS	Oxidative Phosphorylation Complexes
p16	Cyclin-Dependent Kinase Inhibitor 2A or p16INK4a
qPCR	Quantitative Polymerase Chain Reaction
SDHB	CII—Succinate Dehydrogenase Complex Iron Sulfur Subunit B
SOD2	Superoxide Dismutase 2
UQCRC2	CIII—Cytochrome B-C1 Complex Subunit 2
WT	Wild Type
WT-SED	Sedentary WT Mice
