# Supplementary material for: Translational Insights into Exercise-Induced Protective Adaptations in 5XFAD Mice and Middle-Aged Amateur Sportsmen
Source: Antioxidants (Basel). 2026 May 31;15(6):698. doi: 10.3390/antiox15060698 (PMC13295279; doi:10.3390/antiox15060698)
Supplement: Supplementary file 1 [file antioxidants-15-00698-s001.zip › antioxidants-4292226-supplementary materials.pdf]

Supplementary Materials

Supplementary Table:

Table S1. List of target genes and their corresponding TaqMan FAM-labeled probes for real-time qPCR analysis.

Function	Gene symbol	Full name	TaqMan Assay ID number[1]	Species
Antioxidant response	*Aldh2*	Aldehyde dehydrogenase 2	Mm00477469_m1	Mouse
	Cat	Catalase	Mm00437992_m1	Mouse
	Gpx1	Glutathione peroxidase 1	Mm00656767_g1	Mouse
	Nfe2l2	NFE2 like BZIP tanscription factor 2	Mm00477784_m1	Mouse
	Sod2	Superoxide dismutase 2	Mm01313000_m1	Mouse
Epigenetics	*Dnmt1*	DNA methyltransferase (cytosine-5) 1	Mm01151063_m1	Mouse
	Dnmt3a	DNA methyltransferase 3A	Mm00432881_m1	Mouse
	Dnmt3b	DNA methyltransferase 3B	Mm01240113_m1	Mouse
	Hdac1	Histone deacetylase 1	Mm02391771_g1	Mouse
	Hdac3	Histone deacetylase 3	Mm00515916_m1	Mouse
	Hdac5	Histone deacetylase 5	Mm01246076_m1	Mouse
Ubiquin proteasome System	*PSMB5*	Proteasome 20S Subunit Beta 5	Hs00605652_m1	Human
	PSMB6	Proteasome 20S Subunit Beta 6	Hs00382586_m1	Human
	PSMB7	Proteasome 20S Subunit Beta 7	Hs00160607_m1	Human
	PSMB8	Proteasome 20S Subunit Beta 8	Hs00544758_m1	Human
	PSMB9	Proteasome 20S Subunit Beta 9	Hs00160610_m1	Human
	PSMB10	Proteasome 20S Subunit Beta 10	Hs00988194_g1	Human
	UBC	Ubiquitin C	Hs01867132_s1	Human
Senescence	*Cdkn1a*	Cyclin dependent kinase inhibitor 1a	Mm00432448_m1	Mouse
	Cdkn2a	Cyclin dependent kinase inhibitor 2a	Mm00494449_m1	Mouse
	Trp53	Tumor protein p53	Mm01731287_m1	Mouse
	CDKN1A	Cyclin Dependent Kinase Inhibitor 1A	Hs00355782_m1	Human
	CDKN2A	Cyclin Dependent Kinase Inhibitor 2A	Hs00923894_m1	Human
	TP53	Tumor Protein P53	Hs01034249_m1	Human
Reference gene	*Actb*	Actin, beta	Mm02619580_g1	Mouse
	Tbp	TATA box binding protein	Mm00446971_m1	Mouse
	B2M	β-2-Microglobulin	Hs00187842_m1	Human
	PGK1	Phosphoglycerate kinase 1	Hs00943178_g1	Human

Note: [1]Commercial TaqMan FAM-labeled probes (Thermo Fisher Scientific).

Supplementary Figures:

a

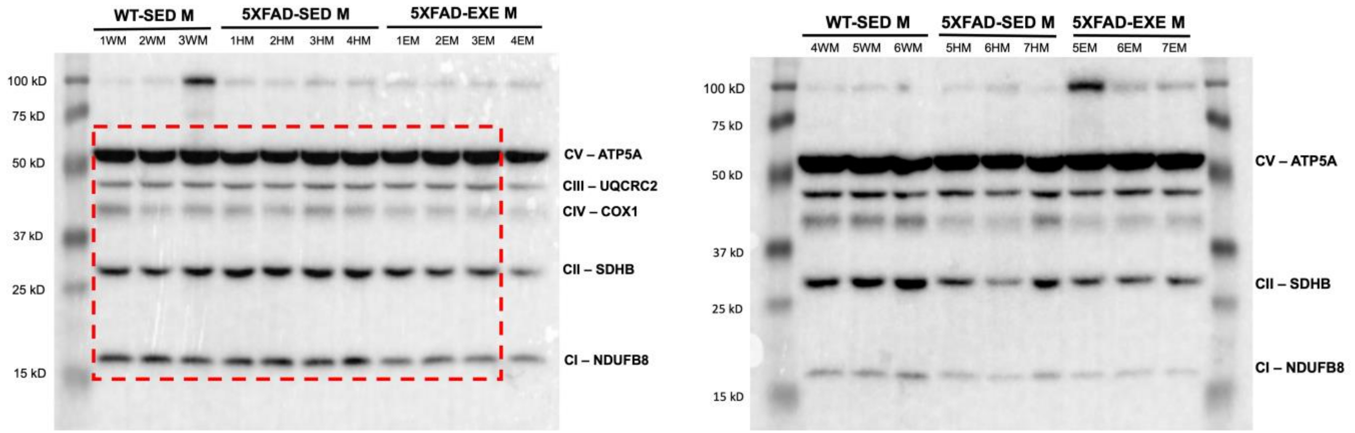

b

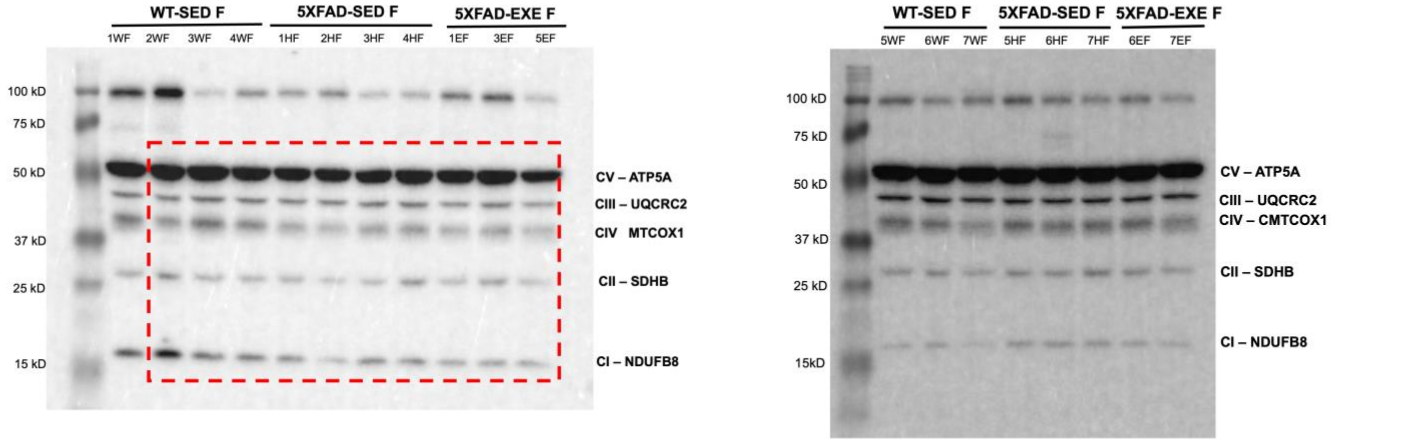

Figure S1. Full western blot membranes probed with anti-OXPHOS antibodies. (**a**) Male samples. (**b**) Female samples. All lanes were included in the quantification. Red-dotted boxes highlight the specific membrane regions cropped and presented in Figure 2. A molecular-weight protein ladder is visible in the leftmost lane for reference.

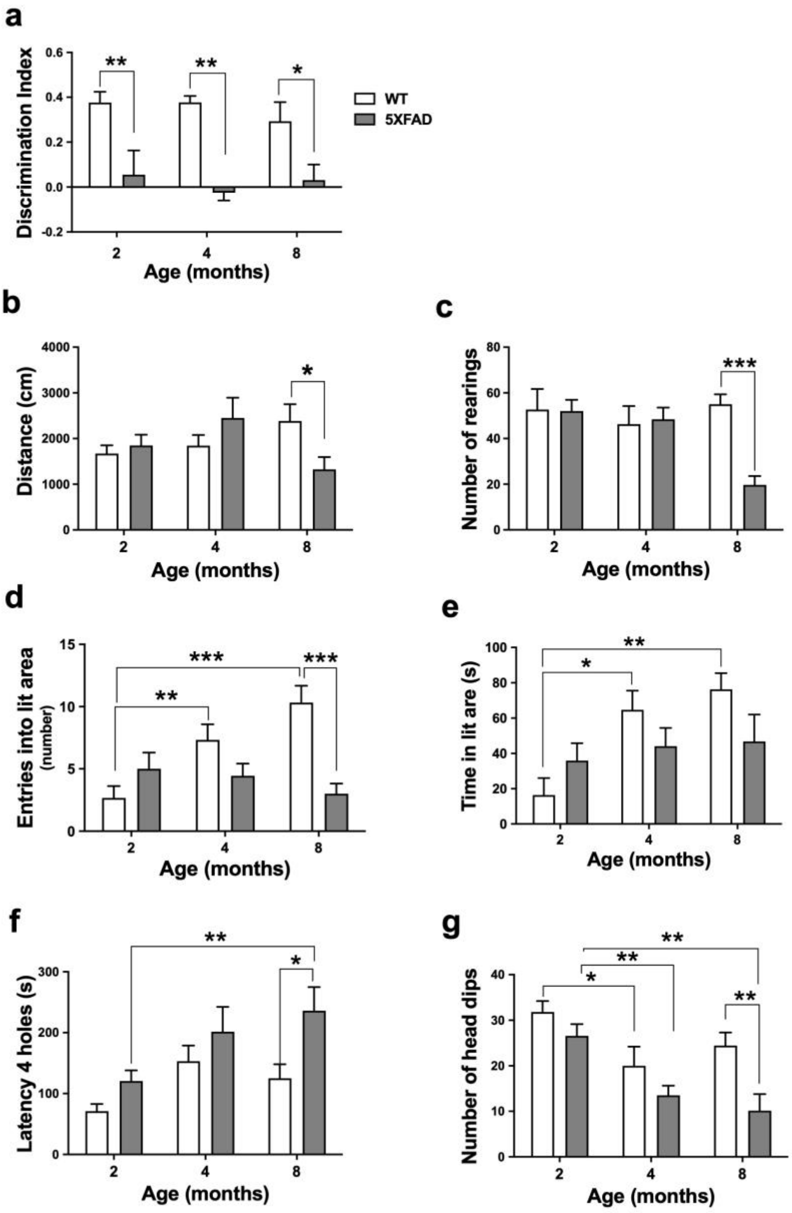

Figure S2. Time course of cognition and general behavior in 5XFAD and WT male mice. (**a**) Novel object recognition test at 2 h. (**b,** c) Horizontal and vertical activity in the open field test. (**d, e**) Number of entries and time into the lit compartment in the light-dark test. (**f, g**) Latency to explore the four holes and total number of head dips in the four-hole board test. Independent groups of male mice aged 2 months (WT, $N = 6$; 5XFAD, $N = 7$) and 4 months (WT, $N = 6$; 5XFAD, $N = 7$) were analyzed together with the corresponding 8-month-old groups from the main study (WT-SED, $N = 6$; 5XFAD-SED, $N = 6$). Values are presented as mean ± SEM. Statistics: Two-way ANOVA showed significant interaction between age and strain in panels **b**, **c**, and **d**; significant age effect in **e**–**g**; and significant strain effect in **a, c, d, f**, and **g** (all $p < 0.05$). Post hoc comparisons *$p < 0.05$, **$p < 0.01$, ***$p < 0.001$.
